# *Bt* rice could provide ecological resistance against nontarget planthoppers

**DOI:** 10.1111/pbi.12911

**Published:** 2018-04-10

**Authors:** Xingyun Wang, Qingsong Liu, Michael Meissle, Yufa Peng, Kongming Wu, Jörg Romeis, Yunhe Li

**Affiliations:** ^1^ State Key Laboratory for Biology of Plant Diseases and Insect Pests Institute of Plant Protection Chinese Academy of Agricultural Sciences Beijing China; ^2^ Research Division Agroecology and Environment Agroscope Zurich Switzerland

**Keywords:** genetically engineered plant, metabolome analysis, host preference, plant‐mediated insect interaction, non‐*Bt* refuge, ecological resistance

## Abstract

Genetically engineered (GE) rice lines expressing Lepidoptera‐active insecticidal *cry* genes from the bacterium *Bacillus thuringiensis* (*Bt*) have been developed in China. Field surveys indicated that *Bt* rice harbours fewer rice planthoppers than non‐*Bt* rice although planthoppers are not sensitive to the produced *Bt* Cry proteins. The mechanisms underlying this phenomenon remain unknown. Here, we show that the low numbers of planthoppers on *Bt* rice are associated with reduced caterpillar damage. In laboratory and field‐cage experiments, the rice planthopper *Nilapavata lugens* had no feeding preference for undamaged *Bt* or non‐*Bt* plants but exhibited a strong preference for caterpillar‐damaged plants whether *Bt* or non‐*Bt*. Under open‐field conditions, rice planthoppers were more abundant on caterpillar‐damaged non‐*Bt* rice than on neighbouring healthy *Bt* rice. GC–MS analyses showed that caterpillar damage induced the release of rice plant volatiles known to be attractive to planthoppers, and metabolome analyses revealed increased amino acid contents and reduced sterol contents known to benefit planthopper development. That Lepidoptera‐resistant *Bt* rice is less attractive to this important nontarget pest in the field is therefore a first example of ecological resistance of *Bt* plants to nontarget pests. Our findings suggest that non‐*Bt* rice refuges established for delaying the development of *Bt* resistance may also act as a trap crop for *N. lugens* and possibly other planthoppers.

## Introduction

Insect‐resistant genetically engineered (GE) plants expressing Cry proteins from the bacterium *Bacillus thuringiensis* (*Bt*) have been widely adopted worldwide; 98.5 million hectares were planted with *Bt* crops in 2016 (ISAAA, 2016). *Bt* plants are efficient in controlling certain target insect pests (Carrière *et al*., 2003; Hutchison *et al*., 2010; National Academies of Sciences, Engineering, and Medicine, 2016; Wu *et al*., 2008) and can lead to a significant decrease in the application of the chemical insecticides which are often necessary in conventional cropping systems (Klümper and Qaim, 2014; National Academies of Sciences, Engineering, and Medicine, 2016). The replacement of such chemical insecticides by *Bt* crops can therefore benefit human health and the environment (Huang *et al*., 2005; Lu *et al*., 2012; Marvier *et al*., 2007; National Academies of Sciences, Engineering, and Medicine, 2016; Shelton *et al*., 2002). The use of *Bt* plants, however, also comes with new challenges. For example, the wide‐scale planting of *Bt* crops can result in increased populations of nontarget pests, probably because of the reduction in insecticide applications in *Bt* crops (Lu *et al*., 2010; Naranjo, 2011).

Rice is an important crop and a primary food source for more than half of the world's population. To control rice insect pests, China has devoted great effort in developing *Bt* rice. Multiple *Bt* rice lines are available for controlling lepidopteran pests, such as the rice stem borers *Chilo suppressalis* Walker (Lepidoptera: Crambidae), *Scirpophaga incertulas* Walker (Lepidoptera: Crambidae) and *Sesamia inferens* Walker (Lepidoptera: Noctuidae) (Chen *et al*., 2011). These *Bt* rice lines mainly express Cry1, Cry2 and Cry9 proteins and have no reported direct toxicity to nontarget species outside the order Lepidoptera (Chen *et al*., 2011; Li *et al*., 2016).

In addition to Lepidoptera, rice planthoppers including *Nilaparvata lugens* Stål and *Sogatella furcifera* Horváth (both Hemiptera: Delphacidae) are primary pests of rice and have frequently experienced outbreaks in China following the adoption of hybrid rice cultivars in the 1980s (Chen *et al*., 2011). Because the planthoppers are not sensitive to the Cry proteins produced by the current *Bt* rice plants (Li *et al*., 2016), they might benefit from the expected reduction in insecticide applications such that the planting of *Bt* rice may lead to planthopper outbreaks similar to those reported for mirid bugs in *Bt* cotton (Lu *et al*., 2010; Naranjo, 2011). Surveys of experimental fields, however, have indicated that planthoppers including *N. lugens* and *S. furcifera* unexpectedly migrate from *Bt* rice fields to adjacent non‐*Bt* rice fields, resulting in low densities in *Bt* rice (Chen *et al*., 2002, 2003, 2007, 2012; Lu *et al*., 2014a,b). Researchers have conducted feeding studies but have found no conclusive evidence that *Bt* rice directly affects planthopper performance (Chen *et al*., 2011; Li *et al*., 2016, 2017), so that the potential mechanisms underlying this phenomenon remain unknown.

The herbivore feeding may alter plant physiology and phenotype and thereby affect the performance, behaviour, and ultimately the population dynamics of other herbivore species is well known. Such plant‐mediated indirect interactions among herbivores may be negative or positive (Poelman and Dicke, 2014; Stam *et al*., 2014). We hypothesize that the migration of rice planthoppers from *Bt* rice fields to adjacent non‐*Bt* rice fields may be caused by an indirect, plant‐mediated interaction involving the lepidopteran target pests of *Bt* rice. To test this hypothesis, we studied the plant‐mediated interactions between the lepidopteran target pest *C. suppressalis* and the major nontarget rice planthopper *N. lugens*. In addition to laboratory and field studies, we used GC–MS and metabolome analyses to elucidate potential chemical mechanisms underlying the interactions between the two insect species.

## Results

### Performance of the lepidopteran target pest on *Bt* and non‐*Bt* rice

When 1st instars of *C. suppressalis* fed on stems cut from rice, the survival rate after 5 days was 87.6% with non‐*Bt* stems and 0.0% with *Bt* stems, which indicates high efficiency of *Bt* rice against neonates. For the 3rd instars, in contrast, the survival rate after 3 days was 83.7% with non‐*Bt* stems and 83.0% with *Bt* stems, and there was no significant difference between the two treatments (χ^*2 *^= 0.008, *df *= 1, *P *=* *0.929). However, the increase in body weight was significantly greater for 3rd instars fed on non‐*Bt* rice plants (increase = 7.80 ± 0.95 mg) than on *Bt* rice plants (increase = 0.57 ± 0.14 mg) (*t *=* *7.80, *df *= 83, *P *<* *0.001). These results show that *Bt* rice plants significantly reduce *C. suppressalis* performance, while the 3rd instar larvae can still cause damage on the plants.

### Preference of *N. lugens* for undamaged and caterpillar‐damaged *Bt* and non‐*Bt* rice

**Figure 1:**
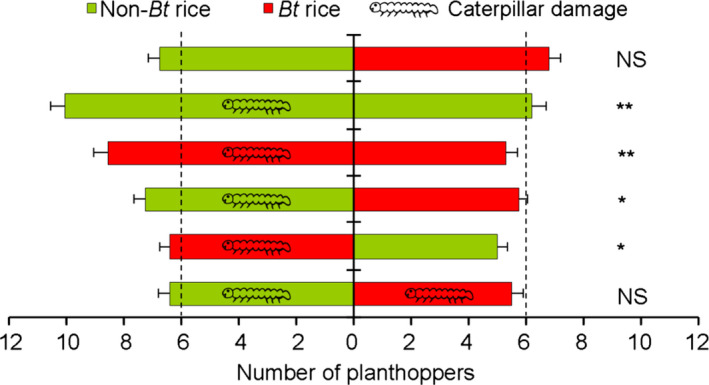
Preference of the rice planthopper *Nilaparvata lugens* for undamaged or caterpillar‐damaged *Bt* or non‐*Bt* rice plants. Caterpillar symbols indicate damage by one 3rd instar of *Chilo suppressalis*. All choice tests were performed with 20–23 replicates, each consisting of a group of 20 *N. lugens*. The number of planthoppers represents the average of daily values based on 7‐day observation periods. Asterisks indicate significant differences: *P <* *0.05, **P <* *0.01; NS indicates no significance (*P* > 0.05) (paired‐sample *t*‐test).

When given a choice between undamaged *Bt* and non‐*Bt* rice plants in laboratory tests, *N. lugens* showed no feeding preference (*t *= −0.15, *df *= 22, *P *=* *0.886) (Figures 1 and S1A). However, the planthoppers exhibited a strong preference for rice plants which were damaged by *C. suppressalis* caterpillars over undamaged rice plants whether they were non‐*Bt* or *Bt* rice (damaged non‐*Bt* versus undamaged non‐*Bt*:* t *=* *4.42, *df *= 19, *P *<* *0.001; damaged *Bt* versus undamaged *Bt*:* t *=* *4.59, *df *= 21, *P *<* *0.001; damaged non‐*Bt* versus undamaged *Bt*:* t *=* *2.51, *df *= 19, *P *=* *0.021; and damaged *Bt* versus undamaged non‐*Bt*:* t *=* *2.67, *df *= 19, *P *=* *0.015) (Figures 1 and S1B–E). When both *Bt* and non‐*Bt* plants were damaged by caterpillars, no feeding preference by the planthoppers was evident (*t *=* *1.43, *df *= 19, *P *=* *0.169) (Figures 1 and S1F). These results demonstrate that planthoppers show no preference for *Bt or* non‐*Bt* rice plants but prefer caterpillar‐damaged over undamaged rice plants.

### Fitness of *N. lugens* feeding on undamaged or caterpillar‐damaged *Bt* and non‐*Bt* rice plants

There was no significant difference in survival rate, nymphal development time or adult body length of *N. lugens* when fed undamaged *Bt* rice, undamaged non‐*Bt* rice, caterpillar‐damaged *Bt* rice or caterpillar‐damaged non‐*Bt* rice (all *P *>* *0.05) (Table 1). The fresh weight of eight‐day‐old *N. lugens* nymphs, however, was much greater on caterpillar‐damaged plants than on undamaged plants, although the difference was significant only for *Bt* plants (*P *=* *0.003) and not for non‐*Bt* plants (*P *>* *0.05) (Table 1). These results show that planthopper fitness is significantly greater on caterpillar‐damaged than on undamaged rice plants.

**Table 1:** Performance of the rice planthopper *Nilaparvata lugens* feeding on *Bt* and non‐*Bt* rice plants which were undamaged or damaged by a single 3rd instar of *Chilo suppressalis*

Parameter	Non‐*Bt* rice undamaged	Non‐*Bt* rice damaged	*Bt* rice undamaged	*Bt* rice damaged
Survival rate^a^	76% (34) a	85% (40) a	82% (38) a	73% (33) a
Eight‐day nymph fresh weight (mg)^b^	0.90 ± 0.04 (141) ab	0.92 ± 0.04 (145) ab	0.83 ± 0.03 (138) b	1.02 ± 0.04 (142) a
Female nymph development (day)^c^	14.62 ± 0.13 (119) a	14.76 ± 0.13 (143) a	14.47 ± 0.12 (151) a	14.44 ± 0.13 (103) a
Male nymph development (day)^c^	14.32 ± 0.13 (115) a	14.45 ± 0.14 (122) a	14.23 ± 0.12 (142) a	14.08 ± 0.12 (142) a
Female adult body length (mm)^b^	3.61 ± 0.03 (54) a	3.61 ± 0.03 (72) a	3.68 ± 0.02 (75) a	3.71 ± 0.03 (47) a
Male adult body length (mm)^b^	2.79 ± 0.02 (48) a	2.80 ± 0.03 (60) a	2.85 ± 0.02 (81) a	2.87 ± 0.01 (79) a

a Chi‐square test with Bonferroni correction (adjusted α = 0.008).

b One‐way ANOVA followed by Tukey's HSD tests for pairwise comparisons.

c Kruskal–Wallis test.

Values are means ± SE, and numbers of specimens tested are indicated in parentheses. Different letters in the same row indicate significant differences (*P* < 0.05).

### Abundance of *N. lugens* on *Bt* and non‐*Bt* rice under field conditions

**Figure 2:**
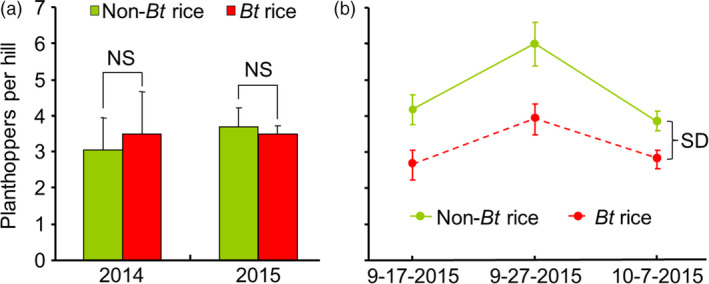
Abundance of the rice planthopper *Nilaparvata lugens* on *Bt* and non‐*Bt* rice plants in field experiments. (a) *N. lugens* were released in field cages (1.5 × 1 × 1 m) containing both *Bt* and non‐*Bt* rice at the heading stage. Plants were surveyed 40 days later. No *Chilo suppressalis* was present in the cages. The experiment was conducted in 2014 with three replicates and in 2015 with four replicates. NS indicates no significant difference (*P *>* *0.05) (Student's *t*‐test). (b) *Bt* and non‐*Bt* rice were planted in 2 × 2 m plots under open‐field conditions and were artificially infested with 15 eggs of *C. suppressalis* per plant at the jointing/heading stage (25–27 August 2015). Naturally occurring planthoppers on *Bt* and non‐*Bt* rice plants were counted on 17 September, 27 September and 7 October 2015. The experiment included seven *Bt* and eight non‐*Bt* plots. SD indicates a significant difference between treatments (RM‐ANOVA;* P *<* *0.01). Values are means ± SE.

Field‐cage experiments were conducted in 2014 and 2015. In the first experiment, undamaged *Bt* rice and non‐*Bt* rice plants were contained together in cages made of insect nets and infested with *N. lugens*. Forty days later, planthopper abundance did not significantly differ on *Bt* versus non‐*Bt* plants (*t *=* *0.68, *df *= 2, *P *=* *0.564 for 2014 and *t *=* *0.34, *df *= 3, *P *=* *0.756 for 2015) (Figure 2a). In the second experiment, *Bt* and non‐*Bt* rice plants in open‐field plots were artificially infested with *C. suppressalis* eggs. After 20, 30 and 40 days, the abundance of naturally occurring *N. lugens* was significantly higher on the caterpillar‐susceptible non‐*Bt* rice than on the *Bt* rice plants (*F*_1,13 _= 16.30, *P *=* *0.001) (Figure 2b). On the last observation date, 82.5% of the non‐*Bt* rice plants but only 6.7% of the *Bt* plants exhibited caterpillar damage. The results from these cage and field experiments demonstrate that planthopper abundance is greater on non‐*Bt* than on *Bt* rice plants in the presence of *C. suppressalis*.

### Rice plant volatiles induced by caterpillar damage

**Figure 3:**
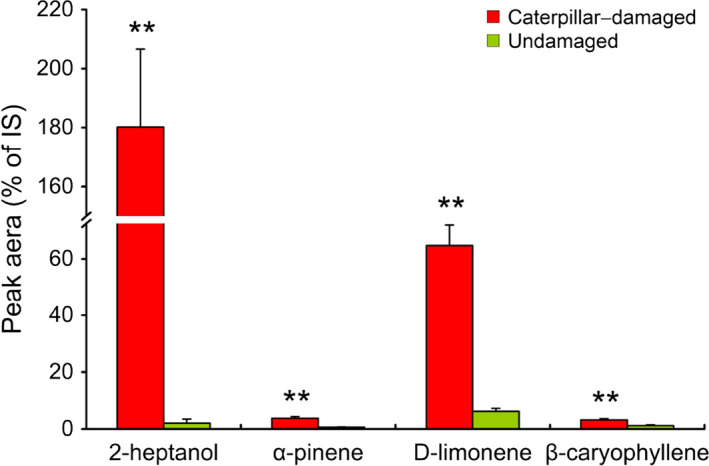
Relative amounts of volatile compounds emitted from caterpillar‐damaged and undamaged non‐*Bt* rice plants. Values are mean percentages ± SE of the peak area of the internal standard (nonyl acetate). Asterisks indicate significant differences between undamaged and caterpillar‐damaged rice plants (Student's *t*‐test, **P <* *0.01). Data were log10(x + 1) transformed before analyses.

A total of 36 compounds were identified in the headspace of non‐*Bt* rice plants damaged by *C. suppressalis* (Table S1). Most of these compounds (55.6%) were significantly increased in response to caterpillar infestation. In addition, several compounds which were not measurable in undamaged plants, such as (E)‐2‐hexenal, β‐pinene and α‐phellandrene, were induced by *C. suppressalis* feeding (Table S1). Among the significantly increased compounds, 2‐heptanol (86 times higher), α‐pinene (10 times higher), D‐limonene (10 times higher) and β‐caryophyllene (three times higher) have been confirmed to be attractive to *N. lugens* in previous studies (Figure 3; Wang *et al*., 2015; Xiao *et al*., 2012; Zhang *et al*., 2014).

### Metabolomic profile of rice plants after damage by caterpillars

**Figure 4:**
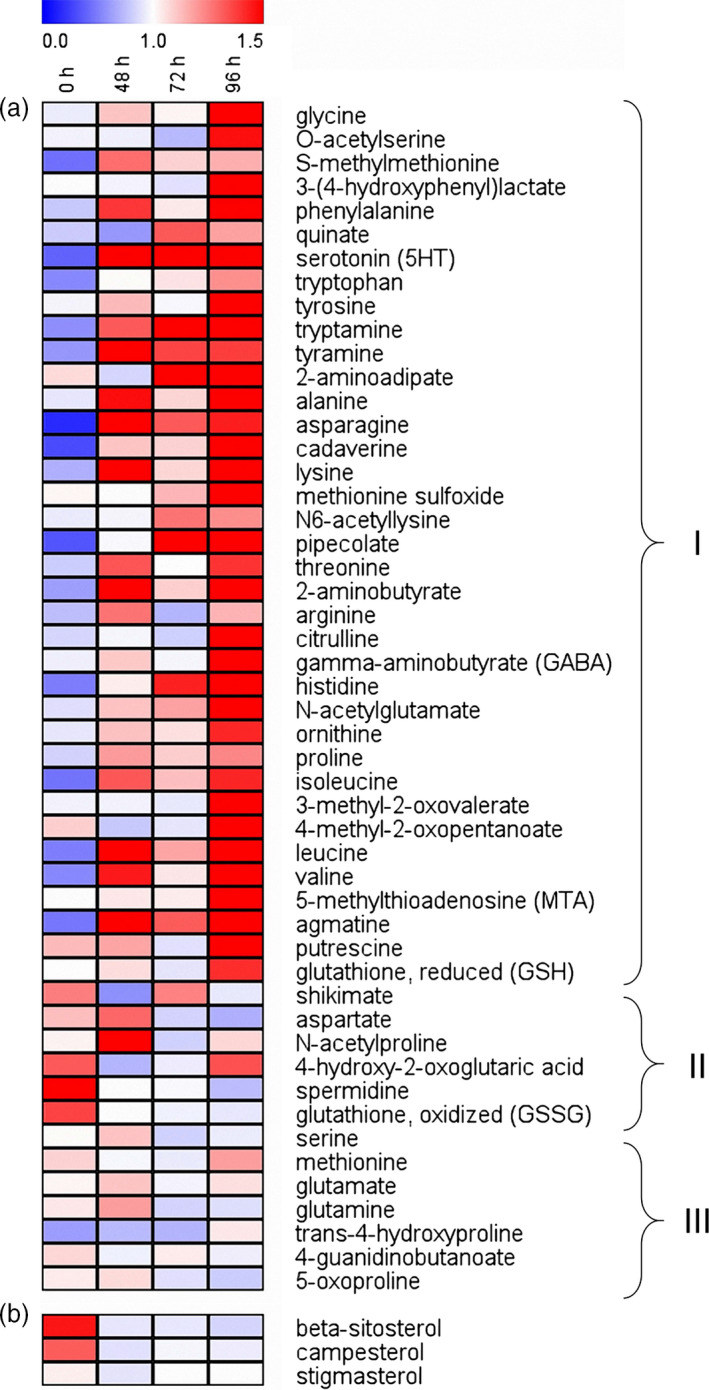
Heatmap presentation of the metabolites in non‐*Bt* rice stems after infestation by one 3rd instar of *Chilo suppressalis* for 0, 48, 72 and 96 h. (a) Amino acids whose contents increased (I), decreased (II) or did not apparently change (III) in rice stems with increasing caterpillar damage. (b) Sterols. Each line in the heatmap represents one metabolite. The colour code indicates the content of each metabolite relative to the median metabolite concentration. Values were generated by normalizing directly on a similar graphic scale by their median values. Red indicates a higher content, and blue indicates a lower content of the metabolite in the sample.

A total of 50 known amino acids were detected and quantified in stems from caterpillar‐damaged and undamaged rice plants. After infestation by *C. suppressalis* larvae, the total concentration of amino acids increased which resulted in a significance at 96 h (Figure S2). More than 74% of the amino acids were increased as a response to caterpillar damage, many already at 48 h. Amino acids such as alanine, asparagine and valine have been shown to stimulate planthopper feeding and development (Sōgawa 1982; Fujita *et al*., 2013; Figure 4a). Three sterols were identified and among them, beta‐sitosterol and campesterol contents tended to decrease, while stigmasterol content showed no apparent change after caterpillar feeding; those sterols have been shown to confer resistance to planthoppers in previous studies (Shigematsu *et al*., 1982; Fujita *et al*., 2013; Figure 4b).

## Discussion

Our laboratory bioassays with *C. suppressalis* larvae showed that the 1st instars cannot survive on *Bt* rice plants and thus do not cause noticeable damage. As a consequence, *Bt* rice is highly resistant to caterpillar feeding in the field (Tu *et al*., 2000). Although the 3rd instars did damage the *Bt* plants in our laboratory bioassays, the damage was substantially lower than on non‐*Bt* plants.

Our laboratory and field experiments indicated that *N. lugens* did not differentiate between healthy *Bt* and non‐*Bt* rice plants but greatly preferred caterpillar‐damaged over undamaged plants, regardless of *Bt* or non‐*Bt*. As a consequence, when *Bt* rice is planted in close vicinity to non‐*Bt* rice, significantly higher caterpillar damage on non‐*Bt* plants will cause planthoppers to move from undamaged *Bt* to damaged non‐*Bt* rice fields, eventually leading to a higher density of planthoppers on non‐*Bt* rice. In fact, this phenomenon had also been observed in a previous large‐scale field study which reported that planthoppers disperse from *Bt* to non‐*Bt* rice fields (Chen *et al*., 2003).

To elucidate the biochemical mechanisms underlying the phenomenon of the planthoppers’ preference to caterpillar‐damaged rice plants, the volatiles emitted by undamaged and caterpillar‐damaged non‐*Bt* rice plants were compared. This analysis was only conducted with non‐*Bt* rice plants as the phenomenon was not related to rice genotype, but was attributed to the different levels of caterpillar damage. Furthermore, in our previous study, we compared the volatile bouquets released by *Bt* and non‐*Bt* rice plants and confirmed that the *Bt* trait had no effect (see table 1 in Liu *et al*., 2015). Our GC–MS analyses revealed that concentrations of the rice volatiles 2‐heptanol, α‐pinene, D‐limonene and β‐caryophyllene were significantly increased by caterpillar damage. Those volatiles have been previously confirmed to be attractive to planthoppers (Wang *et al*., 2015; Xiao *et al*., 2012; Zhang *et al*., 2014). According to a preference–performance relationship often observed for phytophagous insects, the preference of rice planthoppers for caterpillar‐damaged rice plants may indicate a better performance on damaged plants than on undamaged plants (Gripenberg *et al*., 2010). Consistent with this relationship and with findings that host plant selection enables insects to develop on hosts that provide optimal nutrition (Bruce *et al*., 2005), we found that the weight of the planthopper nymphs was greater when fed on caterpillar‐damaged plants than on undamaged plants. Host selection by planthoppers is greatly affected by nutritional compounds and chemical cues, such as amino acids and sterols (Fujita *et al*., 2013; Shigematsu *et al*., 1982; Sōgawa, 1982). Previous studies indicated that the amino acid content in rice plant tissues is positively correlated with the susceptibility of the plants to rice planthoppers (Fujita *et al*., 2013; Sōgawa, 1982). In contrast, sterols, including beta‐sitosterol, stigmasterol and campesterol, have been reported to inhibit *N. lugens* feeding (Shigematsu *et al*., 1982). Our metabolome analyses showed that feeding by *C. suppressalis* larvae induced an increase in the contents of most amino acids and a decrease in the contents of beta‐sitosterol and campesterol in rice. The metabolomics data in combination with the results from the volatile analyses provide a strong explanation of why *N. lugens* prefers to feed on caterpillar‐damaged rice plants. In other words, the results were consistent with the general preference–performance hypothesis (Gripenberg *et al*., 2010) in that *N. lugens* selects caterpillar‐damaged rice over undamaged rice because it performs better on caterpillar‐damaged rice.

Our laboratory bioassays revealed that planthopper survival, developmental time, body weight and body length did not differ on *Bt* rice versus the near‐isogenic non‐*Bt* rice when both kinds of rice plants were not infested with *C. suppressalis*. This is consistent with previous studies (Chen *et al*., 2011; Li *et al*., 2016) and indicates the absence of relevant unintended changes in the *Bt* plants as a side effect of genetic engineering (Ladics *et al*., 2015; Schnell *et al*., 2015).

As noted in the introduction, one concern with *Bt* crops is their increased potential for outbreaks of nontarget pests. Such outbreaks have been observed for hemipteran herbivores, such as leafhoppers, aphids, plant bugs (Miridae) and stink bugs (Pentatomidae) in *Bt* cotton fields in China and the USA (Lu *et al*., 2010; Naranjo, 2011). The population increase in those secondary pests can be linked to the reduction in broad‐spectrum insecticide applications for controlling target lepidopteran pests in *Bt* cotton compared to conventional cotton (Lu *et al*., 2010; Naranjo, 2011). This phenomenon may also be partly explained by plant‐mediated mechanisms. Effective suppression of lepidopteran pests on *Bt* cotton resulted in reduced levels of induced terpenoids, which led to improved performance of cotton aphids (Hagenbucher *et al*., 2013). The current study, however, revealed for the first time that a reduction in the target pest populations on *Bt* plants resulted in a lower density of nontarget pests. Our finding suggests that *Bt* rice plants exhibit ‘ecological resistance’ to planthoppers due to reduced caterpillar damage, although large‐scale field surveys are necessary to further determine the extent of this effect on planthopper populations, as other biotic or abiotic factors will affect the population dynamics of arthropods in the fields. The term ‘ecological resistance’ was used by Von Holle and Simberloff (2005) to describe the different biotic and abiotic properties of an ecosystem that affect the invasion by nonindigenous species. Here, we use the term to demonstrate that the movement of planthoppers from *Bt* rice to non‐*Bt* rice is caused by indirect, plant‐mediated interactions between *C. suppressalis* and *N. lugens*, and not by direct effects of *Bt* rice on the planthopper.

A major challenge for the sustainable production of *Bt* crops is the potential for insects to evolve resistance to the produced *Bt* proteins (Tabashnik and Carrière, 2017; Tabashnik *et al*., 2013; Wan *et al*., 2017). The most commonly practised strategy for delaying the development of *Bt* resistance is the establishment of refuges with non‐*Bt* plants (Gould, 2000; Tabashnik and Carrière, 2017; Tabashnik *et al*., 2013). The establishment of non‐*Bt* rice refuges next to the *Bt* plants may even be required for the commercial planting of *Bt* rice. The current findings suggest that such non‐*Bt* refuges have more than one function, that is in addition to acting as a source of *Bt*‐susceptible caterpillars, they may also act as a trap crop for planthoppers (Shelton and Badenes‐Perez, 2006).

In summary, the current findings suggest that *Bt* rice may provide ecological resistance to planthoppers. Combining the results from available field surveys indicating lower densities of planthoppers in *Bt* than non‐*Bt* rice fields (Chen *et al*., 2002, 2003, 2007, 2012; Lu *et al*., 2014a,b), this finding implies that outbreaks of rice planthoppers may not occur in commercial plantings of *Bt* rice when non‐*Bt* rice is planted with *Bt* rice plants. In addition, our results suggest that the establishment of non‐*Bt* rice refuges may not only delay the evolution of *Bt* resistance in the target pests but may also attract and trap rice planthoppers.

## Materials and methods

### Plants and insects

The transgenic *Bt* rice line Huahui No. 1 and the corresponding nontransformed near‐isoline Minghui63 were used in all experiments. Huahui No. 1 expresses a fused *cry1Ab/Ac* gene under the control of the rice *actinI* promoter. Minghui63 is an elite indica restorer line for cytoplasmic male sterility in China and served as the nontransgenic control in this study. The rice seeds were provided by Prof. Yongjun Lin (Huazhong Agricultural University, Wuhan, China).

*Chilo suppressalis* larvae were obtained from a laboratory colony that had been maintained on an artificial diet for >50 generations with yearly introductions of individuals from natural populations. Specimens of *N. lugens* were obtained from a laboratory colony that had been maintained on conventional rice plants, Taichung Native 1 (TN1), for >5 generations. Both insect colonies were kept at 27 ± 2°C, 70%–80% RH and a photoperiod of 16:8 h (light: dark) at the Institute of Plant Protection, Chinese Academy of Agricultural Sciences, Beijing, China.

### Laboratory studies with caterpillars and planthoppers

#### Plant preparation

Pregerminated seeds of *Bt* and non‐*Bt* rice were simultaneously sown in a glasshouse at 28 ± 2°C with 65 ± 10% RH and a photoperiod of 16:8 h (light: dark). After 15 days, the seedlings were individually transplanted into clay pots (diameter 8 cm, height 10 cm) containing a 3:1 mixture of peat and vermiculite. Plants were watered and supplied with nitrogenous fertilizer (Sakefu, Sino‐Arab Chemical Fertilizer Co., Ltd, Qinhuangdao, China) containing N (20%), P_2_O_5_ (20%), K_2_O (20%) and other microelements every 3 days and were used for the experiments at the jointing stage, 50–60 days after transplanting. In treatments with caterpillar damage, *Bt* or non‐*Bt* rice plants were individually infested with one 3rd instar larva of *C. suppressalis* that had been starved for at least 3 h (referred to as ‘caterpillar‐damaged plants’ or ‘damaged plants’ in this article). Three days later, caterpillars had drilled into the stems of the plants and had caused visible damage. Caterpillars remained in the plants for the duration of the experiments. Plants in the treatments without damage remained uninfested by caterpillar (referred to as ‘undamaged plants’ in this article).

#### Performance of caterpillars on Bt and non‐Bt rice

Experiments were conducted with 1st and 3rd instar *C. suppressalis*. For the 1st instars, six individuals were transferred to a Petri dish (6 cm diameter) containing segments which were cut from the middle part of the main stems of *Bt* or non‐*Bt* rice plants. Thirty‐nine Petri dishes were set up for each treatment. Five days later, the surviving insects were counted in each Petri dish. For the 3rd instars, insects were weighed on an electronic balance (CPA2250, Sartorius AG, Germany, readability = 0.01 mg) and subsequently placed individually on living *Bt* or non‐*Bt* rice plants. After feeding for 3 days, the surviving insects were counted and weighed again. The experiment was initiated with 49 and 53 insects for the non‐*Bt* and the *Bt* treatments, respectively.

#### Host preference of planthoppers

Six choice tests were conducted with *N. lugens*: (i) healthy *Bt* rice plants versus healthy non‐*Bt* rice plants; (ii) damaged non‐*Bt* rice plants versus healthy non‐*Bt* rice plants; (iii) damaged *Bt* rice plants versus healthy *Bt* rice plants; (iv) damaged non‐*Bt* rice plants versus healthy *Bt* rice plants; (v) damaged *Bt* rice plants versus healthy non‐*Bt* rice plants; and (vi) damaged non‐*Bt* rice plants versus damaged *Bt* rice plants. For each choice test, the main stems of a pair of rice plants were contained in a cylindrical plastic tube (diameter 8.0 cm, length 19.0 cm) (Figure S3). Twenty 3rd instars of *N. lugens* were released in the middle of the cylindrical tube, and the number of planthoppers per plant was recorded for 7 consecutive days. By the last day, most *N. lugens* had developed into adults, and *C. suppressalis* had developed into the 5th instars or prepupae. Each choice test was repeated 20–23 times (replicates).

#### Performance of planthoppers

Two bioassays were conducted with four treatments, namely undamaged and caterpillar‐damaged *Bt* and non‐*Bt* rice plants. The first bioassay was for determining the survival rate of *N. lugens*, and the second was for determining other life table parameters of developmental time, weight and body length. The caterpillar‐damaged rice plants were treated as described above. The procedure for both bioassays was the same. The main stem of a single living plant from each treatment was enclosed in a plastic cylindrical tube (diameter 8.0 cm, height 9.5 cm; Figure S4). One first‐instar nymph of *N. lugens* was introduced into the cylindrical tube. The first bioassay started with 33–40 individuals for each damage × plant treatment. After 18 days feeding, the survival rate of planthoppers in each treatment was recorded. The second started with 135–195 individuals for each treatment. After 8 days of feeding, the living nymphs were weighed on an electronic balance (CPA2250, Sartorius AG, Germany, readability = 0.01 mg). Planthoppers developing into adults were sexed, and their body length was determined; the adults were photographed with a digital camera (DP73, Olympus, Japan) mounted on a microscope (SZX7, Olympus, Japan).

### Experiments under field conditions

#### Cage experiment

To clarify whether *N. lugens* differentiates between healthy *Bt* and non‐*Bt* rice plants, a cage study was carried out in a field near Langfang City (39.5°N, 116.4°E), China. *Bt* and non‐*Bt* rice were sown in a seeding bed on 29 April 2014. The plants were transplanted into clay pots (three plants per pot, similar to a hill in common rice‐growing practice) (diameter 20 cm, height 18 cm) on 12 June, when the plants were at the tillering stage. All plants were covered with a cage made of 80‐mesh nylon nets to prevent insect infestation. The plants were cultivated according to the common local agricultural practices but without pesticide sprays. On 1 August, when the rice plants were at the jointing stage, nine *Bt* and nine non‐*Bt* rice pots were moved into a cage (1.5 m × 1.0 m × 1.0 m) made of 80‐mesh nylon nets. *Bt* plants (3 × 3) were located at one end of the cage, and non‐*Bt* plants were located at the other end with 30 cm of free space in the middle. Three replicate cages were used. One month later (1 September), when rice plants were at the heading stage, approximately 100 *N. lugens* (mixed stages) were released into each cage by placing an open plastic box containing the insects in the centre of the cage. On 10 October, the number of *N. lugens* per pot (hill) was determined. For planthopper counting, a white tray containing a small volume of water was held at a 45° angle to the ground near the base of each pot, and the plants were then vigorously beaten by hand 15 times. The nymphs and adults that fell into the tray were visually counted. The experiment was repeated in 2015 with four replicate cages.

#### Open‐field experiment

To study the abundance of planthoppers in a more realistic setting, a field experiment was conducted in 2 m × 2 m plots with 1 m of bare soil between plots. Eight plots were planted with *Bt* rice, and another eight were planted with non‐*Bt* rice in a randomized block design. Each plot contained 64 hills of rice plants (three plants per hill). *Bt* rice and non‐*Bt* rice seeds were sown on 29 April 2015, and seedlings were transplanted into the plots on 11 June. A 1‐m strip of conventional rice (Minghui86) surrounded the whole experimental field. The plants were cultivated according to local agricultural practices but without pesticide sprays. On 25–27 August, when the plants were at the jointing/heading stage, each *Bt* and non‐*Bt* plant was artificially infested with 15 eggs of *C. suppressalis*. On 17 September, 27 September and 7 October, naturally occurring *N. lugens* were counted on five randomly selected hills of plants in each plot using the same method described above. To estimate the percentage of *Bt* and non‐*Bt* rice plants damaged by *C. suppressalis*, five hills of rice plants were randomly selected from each plot after the final assessment of planthopper numbers; the stems were split, damage by *C. suppressalis* larvae was scored (yes/no) and the percentage of damaged plants was calculated. One *Bt* plot was excluded from analyses because of poor plant growth.

### Rice plant volatiles induced by caterpillar damage

Non‐*Bt* rice plants at the jointing stage were damaged by the 3rd instar larvae of *C. suppressalis* for 24 h using the method described above, or remained undamaged. Volatiles were collected from damaged and undamaged rice plants using the method described by Liu *et al*. (2015), with the exception that nonyl acetate was used as an internal standard. For each treatment, 9–10 collections (from 9 to 10 replicate bottles) were made. Volatiles collected with Super Q traps were extracted with 600 μL of methylene chloride. As an internal standard, 2000 ng of nonyl acetate in 10 μL of methylene chloride was added to the samples. All extracts were stored at –30°C for further analyses.

A gas chromatography mass spectrometry (GC–MS) QP2010 SE (Shimadzu, Kyoto, Japan) equipped with an Rtx‐5 MS capillary column (30.0 m × 0.20 mm id, 0.25 μm film thickness) (Shimadzu, Kyoto, Japan) was used to separate and detect the collected rice volatiles. The injector temperature was 250°C, and splitless mode was used with a splitless time of 1 min. Helium was used as carrier gas with a linear velocity of 34.2 mL/s. The temperature of the detector interface and the ion source remained at 280°C and 230°C, respectively. A 1‐μL volume of each sample was injected. The GC oven temperature was programmed as follows: 40°C for 2 min, followed by increases to 250°C at 6°C/min and maintenance at 250°C for 2 min. The mass spectrometer was operated in scan mode with a mass range of 33–330 m/z and was in an electron impact ionization mode (EI) at 70 Ev. Volatiles were identified by comparing their retention times and mass spectra with authentic standard compounds. If standards were unavailable, tentative identifications were made based on referenced mass spectra available from the NIST14 and NIST14s libraries (Scientific Instrument Services, Inc., Ringoes, NJ). All data were analysed using GCMS solution version 4.20 (Shimadzu, Kyoto, Japan, Shimadzu).

### Metabolomic profile of rice plants after damage by caterpillars

The metabolic data analysed in this study were originally collected in a previous study using the same non‐*Bt* rice line (Minghui63) and treatments (Liu *et al*., 2016). In brief, stems samples of non‐*Bt* rice plants which were undamaged or had been damaged by *C. suppressalis* for 48, 72 and 96 h were analysed using ultra‐high performance liquid chromatography–tandem mass spectroscopy (UHPLC–MS) and gas chromatography–mass spectrometry (GC–MS) platforms (Metabolon Inc., Durham, NC) (Liu *et al*., 2016). The quantitative values of metabolites were derived from integrated raw detector counts of the mass spectrometers, and those with great variations due to instrument interday tuning differences were normalized directly on a similar graphical scale, with the normalized intensities scaled by their median values. Among 151 metabolites identified in rice plants during the 96 h of caterpillar feeding (Liu *et al*., 2016), only the amino acids and sterols were retrieved for further analysis in the current study, because they were reported to be most relevant to the interaction between rice plants and planthoppers (Fujita *et al*., 2013; Sōgawa, 1982).

### Data analysis

All data were checked for normality and equality of variances before statistical analysis. Chi‐square tests were conducted to compare survival rates, and Student's *t*‐tests were used to compare the body weight increases of the *C. suppressalis* larvae that fed on *Bt* and non‐*Bt* rice stems. Paired *t*‐tests were conducted to compare the planthopper feeding choices in the laboratory and the densities on *Bt* and non‐*Bt* rice plants in the field‐cage experiment. In the open‐field experiment, the mean densities of *N. lugens* on *Bt* and non‐*Bt* rice were compared using repeated‐measures (RM) ANOVA. For nymphal body weight and body length, one‐way ANOVAs were carried out followed by Tukey's HSD tests. Chi‐square tests were used to compare the survival rates of planthoppers, and Bonferroni corrections were applied for the six pairwise comparisons, resulting in an adjusted α of 0.008. Kruskal–Wallis tests were conducted for nymphal development time. Dunnett's tests were conducted for comparing the total amount of amino acids in rice stems that had been damaged by *C. suppressalis* larvae for 24, 72 or 96 h relative to the undamaged control. All statistical analyses were conducted using the software package SPSS (version 20; SPSS, Inc., Chicago, IL).

## Author contributions

Y.L. conceived the idea. Y.L., X.W. and J.R. designed the research; X.W. and Q.L. performed research; Y.L., X.W., M.M., J.R. and Y.P. analysed the data; and all authors were involved in writing the manuscript.

## Conflict of interest

The authors declare that they have no conflict of interests.

## Supporting information


**Figure S1** Preference of the rice planthopper *Nilaparvata lugens* for undamaged or caterpillar‐damaged *Bt* or non‐*Bt* rice plants during 7 days.
**Figure S2** Abundance of total amino acids in rice stems after infestation by one 3rd instar of *Chilo suppressalis* for 0, 48, 72 and 96 h.
**Figure S3** Experimental apparatus for testing the feeding preference of the rice planthopper *Nilaparvata lugens* for undamaged or caterpillar‐damaged *Bt* or non‐*Bt* rice plants.
**Figure S4** Experimental apparatus for testing the performance of individual rice planthoppers *Nilaparvata lugens* feeding on undamaged or caterpillar‐damaged *Bt* or non‐*Bt* rice plants.
**Table S1** Volatile compounds collected from the headspace of non‐*Bt* rice plants that were undamaged or damaged by *Chilo suppressalis* larvae for 24 h.
