# Supplementary material for: Bt rice could provide ecological resistance against nontarget planthoppers
Source: Plant Biotechnol J. 2018 Apr 10;16(10):1748–55. doi: 10.1111/pbi.12911 (PMC6131420; doi:10.1111/pbi.12911)
Supplement: Supplementary file 1 — Figure S1 Preference of the rice planthopper Nilaparvata lugens for undamaged or caterpillar‐damaged Bt or non‐Bt rice plants during 7 days. Figure S2 Abundance of total amino acids in rice stems after infestation by one 3rd instar of Chilo suppressalis for 0, 48, 72 and 96 h. Figure S3 Experimental apparatus for testing the feeding preference of the rice planthopper Nilaparvata lugens for undamaged or caterpillar‐damaged Bt or non‐Bt rice plants. Figure S4 Experimental apparatus for testing the performance of individual rice planthoppers Nilaparvata lugens feeding on undamaged or caterpillar‐damaged Bt or non‐Bt rice plants. Table S1 Volatile compounds collected from the headspace of non‐Bt rice plants that were undamaged or damaged by Chilo suppressalis larvae for 24 h. [file PBI-16-1748-s001.doc]

**Supplementary Figures**

**
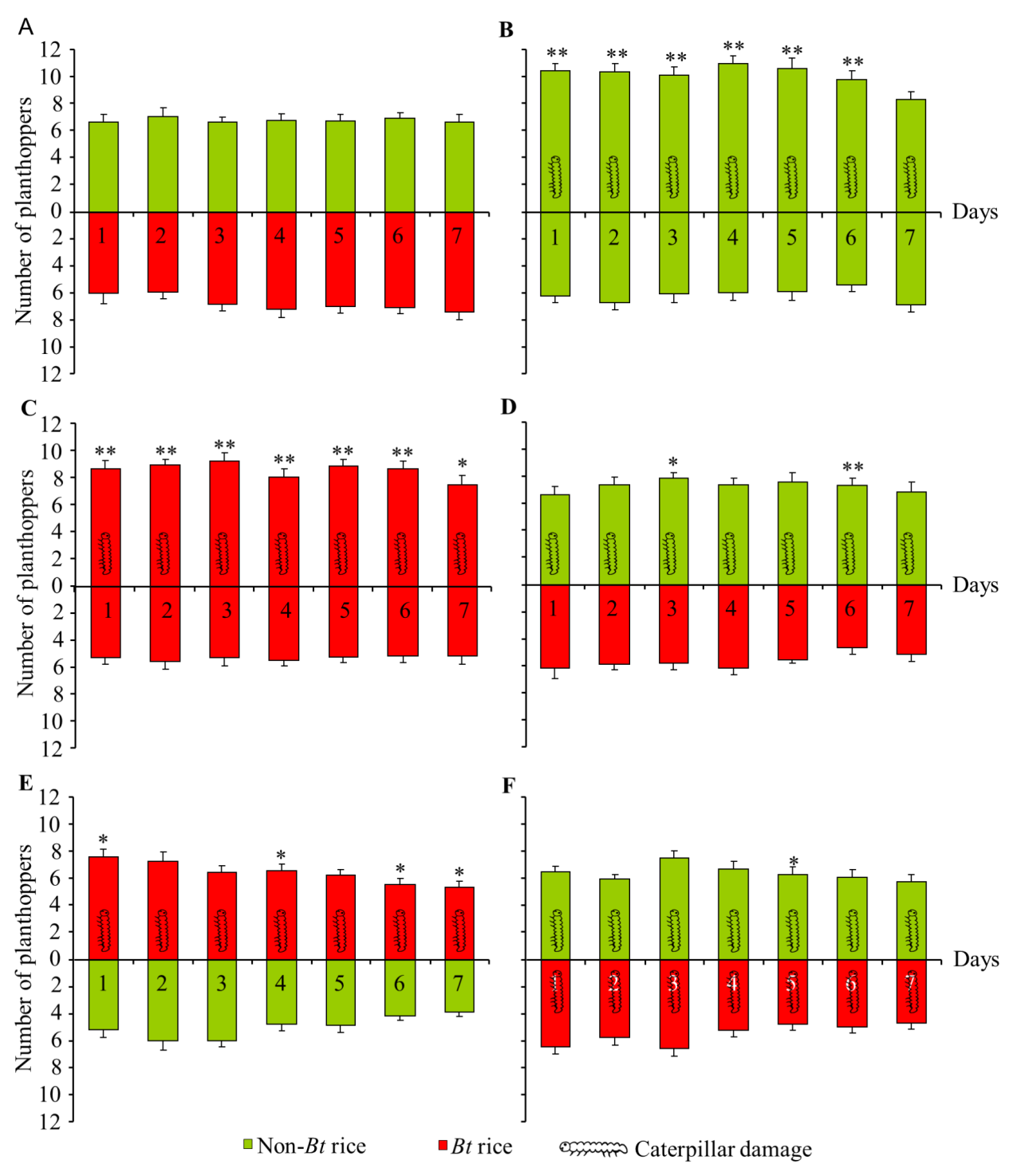
**

**Figure S1.** P**reference of the rice planthopper *Nilaparvata lugens* for undamaged or caterpillar-damaged *Bt* or non-*Bt* rice plants during 7 days.** The caterpillar symbol indicates damage by a single 3^rd^ instar of *Chilo suppressalis*. Each choice test contained 20-23 replicates with a group of 20 *N. lugens* per replicate. The rice plants compared were (a) undamaged *Bt* *vs*. undamaged non-*Bt*; (b) damaged non-*Bt* *vs*. undamaged non-*Bt*; (c) damaged-*Bt* *vs*. undamaged *Bt*; (d) damaged non-*Bt* *vs*. undamaged *Bt*; (e) damaged *Bt* *vs*. undamaged non-*Bt*; and (f) damaged non-*Bt* *vs*. damaged *Bt*. Asterisks indicate a significant difference within a choice test: **P* < 0.05, ** *P* < 0.01.

**
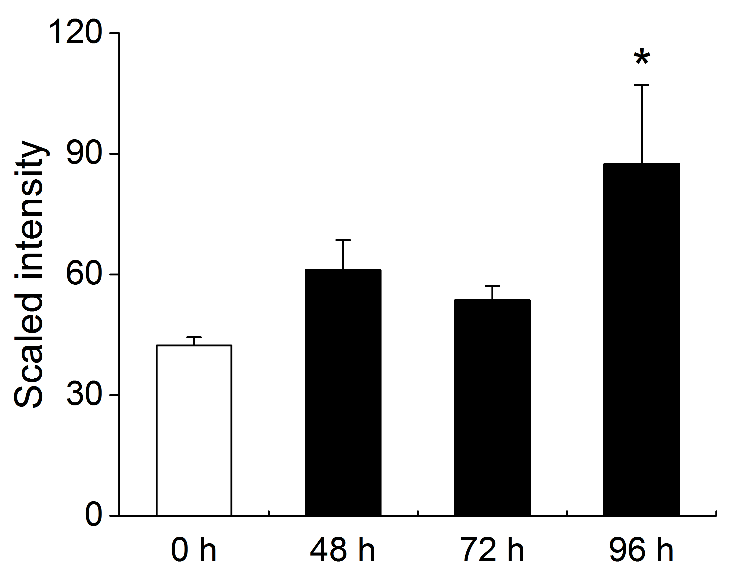
**

**Figure S2. Abundance of total amino acids in rice stems after infestation by one 3^rd^ instar of *Chilo suppressalis* for 0 h, 48 h, 72 h and 96 h.** Values are means ± SE (n=10). The asterisk indicates a significant difference (*P* = 0.014) compared with the uninfected control (one-way analysis of variance with Dunnett’s test).

**
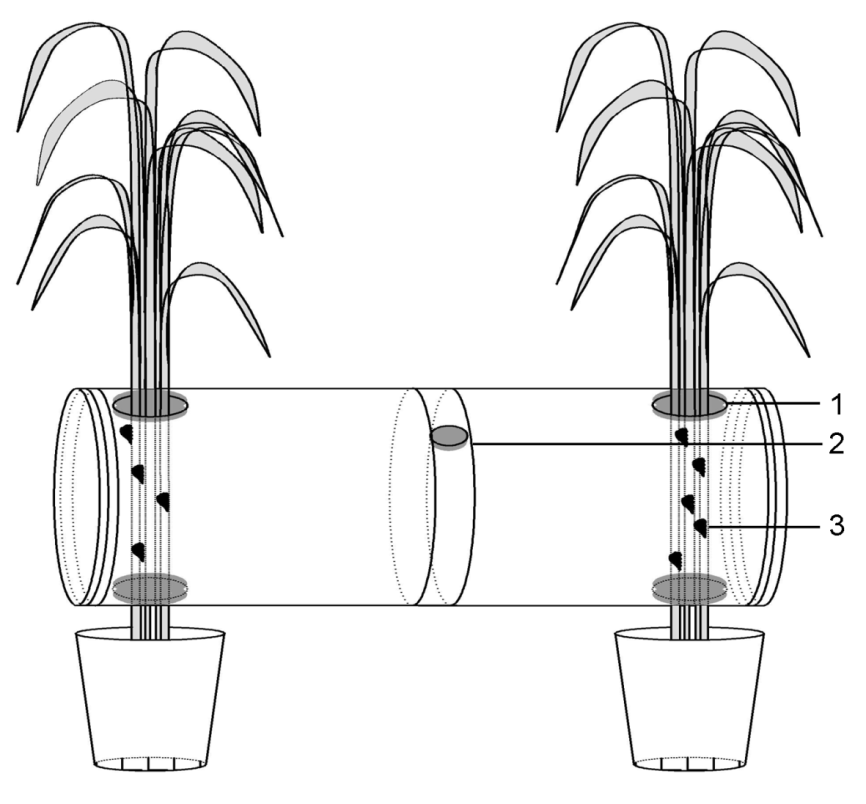
**

**Figure S3. Experimental apparatus for testing the feeding preference of the rice planthopper *Nilaparvata lugens* for undamaged or caterpillar-damaged *Bt* or non-*Bt* rice plants.** The planthoppers were contained in a cylindrical plastic tube with a diameter of 8.0 cm and a length of 19.0 cm. (1) Hole with sponge rubber plug; (2) hole for releasing the planthoppers; (3) planthoppers sitting on rice tillers.

**
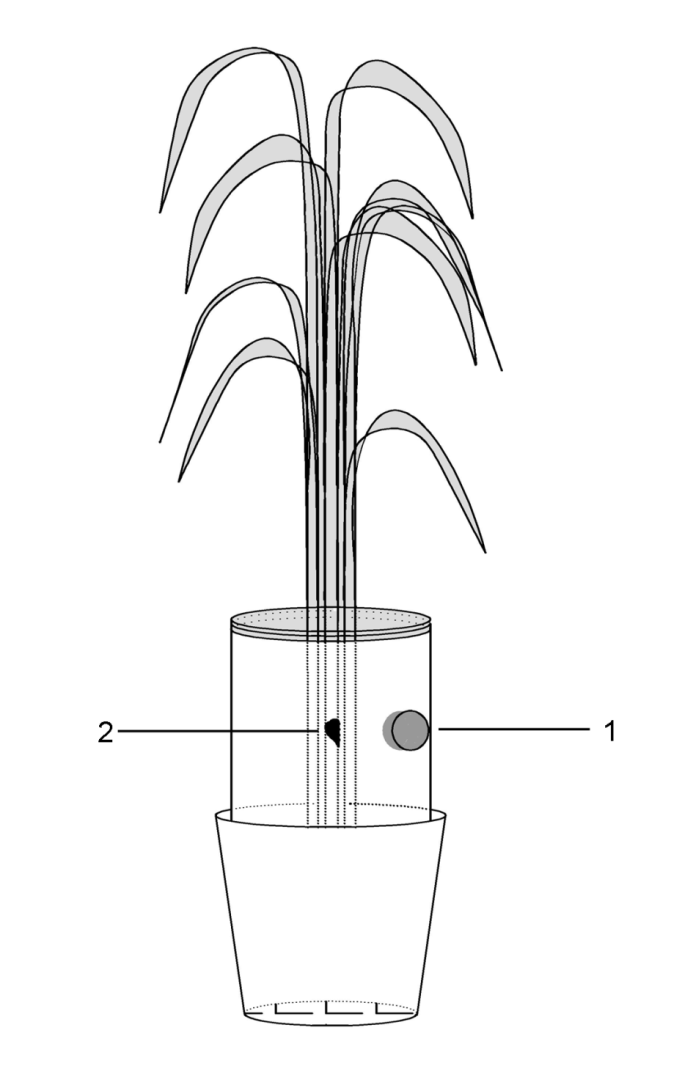
**

**Figure S4. Experimental apparatus for testing the performance of individual rice planthoppers (*Nilaparvata lugens*) feeding on undamaged or caterpillar-damaged *Bt* or non-*Bt* rice plants.** Each planthopper was contained in a cylindrical plastic tube with a diameter of 8.0 cm and a length of 9.5 cm. (1) Hole for releasing the planthopper; (2) planthopper sitting on rice tiller.

**Supplementary Table**

**Table S1. Volatile compounds collected from the headspace of non-*Bt* rice plants that were undamaged or damaged by *Chilo suppressalis* larvae for 24 h**. Values are the mean percentages ± SE of the peak area of the internal standard (nonyl acetate). Asterisks indicate significant differences between undamaged and caterpillar-damaged rice plants according to the Student’s *t*-test (* *P* < 0.05, ** *P* < 0.01 ). Data were log_10_(x + 1) transformed before analyses.

| No. | Compound | Retention time | Undamaged (N=9) | Caterpillar-damaged (N=10) |
| --- | --- | --- | --- | --- |
| 1 | (E)-2-hexenal | 6.52 | 0 ± 0 | 9.82 ± 4.43 |
| 2 | 3-hexen-1-ol | 6.58 | 1.07 ± 0.84 | 3.45 ± 3.45 |
| 3 | 2-heptanone | 7.45 | 0.31 ± 0.19 | 246.66 ± 47.04 ** |
| 4 | 2-heptanol | 7.78 | 2.09 ± 1.39 | 180.17 ± 26.44 ** |
| 5 | α-thujene | 8.38 | 0.36 ± 0.05 | 3.51 ± 0.34 ** |
| 6 | α-pinene | 8.61 | 0.65 ± 0.09 | 3.77 ± 0.61 ** |
| 7 | sabenene | 9.66 | 0.14 ± 0.11 | 1.17 ± 0.2 ** |
| 8 | β-pinene | 9.79 | 0 ± 0 | 0.37 ± 0.1 |
| 9 | β-myrcene | 10.12 | 0.51 ± 0.17 | 4.49 ± 0.63 ** |
| 10 | α-phellandrene | 10.55 | 0 ± 0 | 0.51 ± 0.19 |
| 11 | D-limonene | 11.23 | 6.21 ± 1.07 | 64.74 ± 7.21 ** |
| 12 | γ-terpinene | 12.02 | 0.42 ± 0.14 | 2.98 ± 0.58 ** |
| 13 | (E)-2-heptenyl acetate | 12.26 | 0 ± 0 | 8.48 ± 2.08 |
| 14 | (+)-4-carene | 12.71 | 0 ± 0 | 1.8 ± 0.11 |
| 15 | 2-nonanone | 12.85 | 0.45 ± 0.17 | 89.21 ± 21.84 ** |
| 16 | benzoic acid, methyl ester | 12.97 | 0 ± 0 | 20.48 ± 4.63 |
| 17 | linalool | 13.10 | 22.3 ± 6.9 | 159.84 ± 30.16 ** |
| 18 | (E)-DMNT ^a^ | 13.42 | 2.16 ± 0.55 | 8.15 ± 1.08 ** |
| 19 | methyl salicylate | 15.53 | 3.26 ± 0.32 | 46.9 ± 9.83 ** |
| 20 | 2-undecanone | 17.84 | 0 ± 0 | 18.02 ± 3.57 |
| 21 | α-copaene | 19.83 | 1.43 ± 0.21 | 4.88 ± 0.48 ** |
| 22 | β-caryophyllene | 20.89 | 1.19 ± 0.34 | 3.17 ± 0.44 ** |
| 23 | γ-muurolene | 21.99 | 0 ± 0 | 1.53 ± 0.53 |
| 24 | β-copaene | 22.13 | 0 ± 0 | 3.22 ± 0.95 |
| 25 | (E)-β-ionone | 22.08 | 0 ± 0 | 1.99 ± 1.03 |
| 26 | 2-tridecanone | 22.30 | 0.21 ± 0.21 | 34.81 ± 6.17 ** |
| 27 | β-bsabolene | 22.63 | 0.09 ± 0.09 | 0.82 ± 0.37 |
| 28 | γ-cadinene | 22.79 | 0 ± 0 | 1 ± 0.18 |
| 29 | δ-cadinene | 22.87 | 0 ± 0 | 3.1 ± 0.42 |
| 30 | cis-calamenene | 22.96 | 0 ± 0 | 1.73 ± 0.42 |
| 31 | (E)-γ-bisabolene | 23.02 | 0 ± 0 | 1.04 ± 0.27 |
| 32 | cubenene | 23.17 | 0.28 ± 0.28 | 3.08 ± 0.2 ** |
| 33 | (E)-nerolidol | 23.70 | 0.84 ± 0.29 | 13.12 ± 2.85 ** |
| 34 | (E, E)-TMTT ^b^ | 23.95 | 0.3 ± 0.21 | 1.3 ± 0.34 * |
| 35 | germacrene D-4-ol | 24.07 | 0.2 ± 0.11 | 1.15 ± 0.27 ** |
| 36 | α-cadinol | 25.60 | 0.13 ± 0.13 | 1.63 ± 0.37 ** |

^a^ DMNT, (E)-4,8-dimethylnona-1,3,7-triene.

^b^ TMTT, (3E,7E)-4,8,12-trimethyltrideca-1,3,7,11-tetraene.
